# Asymmetric distribution of enlarged perivascular spaces in centrum semiovale may be associated with epilepsy after acute ischemic stroke

**DOI:** 10.1111/cns.13786

**Published:** 2022-01-03

**Authors:** Nian Yu, Benjamin Sinclair, Lina Maria Garcia Posada, Zhibin Chen, Qing Di, Xingjian Lin, Scott Kolbe, Gernot Hlauschek, Patrick Kwan, Meng Law

**Affiliations:** ^1^ Department of Neurology The Affiliated Nanjing Brain Hospital of Nanjing Medical University Nanjing China; ^2^ Department of Neurology Royal Melbourne Hospital Melbourne Vic. Australia; ^3^ Department of Radiology Alfred Hospital Melbourne Vic. Australia; ^4^ Department of Neuroscience Monash University Melbourne Vic. Australia; ^5^ Department of Neurology Alfred Hospital Melbourne Vic. Australia; ^6^ National Centre for Epilepsy Division of Clinical Neuroscience Oslo University Hospital Oslo Norway; ^7^ Department of Medicine University of Melbourne Melbourne Vic. Australia; ^8^ Department of Neurological Surgery University of Southern California Los Angeles California USA

**Keywords:** asymmetric index, centrum semiovale, enlarged perivascular space, epileptogenesis, post‐stroke epilepsy

## Abstract

**Objective:**

To investigate the factors influencing enlarged perivascular space (EPVS) characteristics at the onset of acute ischemic stroke (AIS), and whether the PVS characteristics can predict later post‐stroke epilepsy (PSE).

**Methods:**

A total of 312 patients with AIS were identified, of whom 58/312 (18.6%) developed PSE. Twenty healthy participants were included as the control group. The number of PVS in the basal ganglia (BG), centrum semiovale (CS), and midbrain (MB) was manually calculated on T_2_‐weighted MRI. The scores and asymmetry index (AI) of EPVS in each region were compared among the enrolled participants. Other potential risk factors for PSE were also analyzed, including NIHSS at admission and stroke etiologies.

**Results:**

The EPVS scores were significantly higher in the bilateral BG and CS of AIS patients compared to those of the control group (both *p* < 0.01). No statistical differences in EPVS scores in BG, CS, and MB were obtained between the PSE group and the nonepilepsy AIS group (all *p* > 0.01). However, markedly different AI scores in CS were found between the PSE group and the nonepilepsy AIS group (*p* = 0.004). Multivariable analysis showed that high asymmetry index of EPVS (AI≥0.2) in CS was an independent predictor for PSE (OR = 3.7, 95% confidence interval 1.5–9.1, *p* = 0.004).

**Conclusions:**

Asymmetric distribution of EPVS in CS may be an independent risk factor and a novel imaging biomarker for the development of PSE. Further studies to understand the mechanisms of this association and confirmation with larger patient populations are warranted.

## Graphical abstract

Higher asymmetry index scores of EPVS in CS are found in post‐stroke epilepsy patients. An asymmetric distribution of EPVS in CS is an independent risk factor or a novel imaging biomarker for post‐stroke epilepsy.

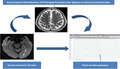

## 1 INTRODUCTION

Acute ischemic stroke (AIS) is one of the most common causes of acquired epilepsy in adults.^1^, ^2^ Many studies have attempted to identify the clinical risk factors for post‐stroke epilepsy (PSE) and to develop prognostic tools, including the PSEiCARe^3^ and SeLECT scores.^4^ However, discovering biomarkers of epileptogenesis after stroke still faces many fundamental challenges. The causes and influential factors of PSE are multifactorial, not only just limited to the processes of stroke or post‐stroke mechanisms but also the prevailing condition of the brain before the stroke.^5^, ^6^

Perivascular spaces (PVSs) are interstitial fluid‐filled cavities surrounding the small penetrating blood vessels^7^ in the brain and are believed to play an important role in glymphatic drainage of waste clearance and maintaining tissue homeostasis.^8^ There is emerging evidence that enlarged PVSs (EPVSs), indicative of dysfunction of glymphatic drainage,^9^ are a feature of brain disorders including small vessel disease,^10^ cognitive impairment,^11^ multiple sclerosis,^12^ and Parkinson's disease.^13^ It has been reported that 98.8% of AIS patients had observable EPVS when scanned within the first 7 days after stroke.^14^ EPVSs were found in the hippocampi of patients with temporal lobe epilepsy^15^, ^16^ and were more frequently observed in the epileptogenic cerebral hemisphere.^17^ This asymmetric distribution of EPVS has been reported in patients with post‐traumatic epilepsy.^18^

These studies suggest that the asymmetric distribution of EPVS may be an imaging biomarker for the development of PSE, yet the specific relationship remains uncertain. The current study aimed to identify the risk factors for EPVS in AIS and to examine whether the EPVS was associated with the development of epilepsy after stroke. We hypothesized that there would be a direct relationship between the number of EPVS detected by MRI during the acute stage of ischemic stroke and the risk of developing PSE.

## 2 PARTICIPANTS AND METHODS

### 2.1 Study design

This is a case‐control study. All patients admitted to the Nanjing Brain Hospital of Nanjing Medical University from Jan 1, 2018, to Oct 31, 2019, with AIS were screened (*n* = 1,465). Patients were followed up for at least 1 year post stroke or until the first occurrence of unprovoked seizures, whichever is sooner. This time point was chosen based on the observation that the incidence of late seizures is highest during the first year and typically peaks around 6–12 months after stroke.^1^, ^19^, ^20^ Age‐ and sex‐matched healthy volunteers were selected as controls. Only AIS patients and healthy controls who had undergone MRI brain with appropriate sequences were included for PVS analysis.

This study was approved by the medical ethics committee of the Nanjing Brain Hospital. All participants or their family members or authorized legal representatives provided written informed consent.

### 2.2 Participants and groups

The inclusion criteria were as follows: (1) any type of AIS patient; (2) MRI obtained within 2 weeks from the stroke onset; and (3) over 16 years old. Individuals were excluded based on the following criteria: (1) history or family history of seizures or epilepsy; (2) history of mental illness, CNS infectious diseases, neurological immune disorders, metabolic disorders, febrile symptomatic seizures, and alcohol/drug withdrawal or intoxication; (3) previous neurosurgery, brain tumor, or traumatic brain injury; (4) patients who were not fit to receive or refused MRI scans at the stage of stroke onset; (5) brain MRI showing one of the following abnormalities: contusion, intracranial hemorrhage, neoplastic lesion, infectious or inflammatory lesion, or hydrocephalus; (6) quality of MRI too poor for evaluation (eg, due to motion artifact). Finally, 312 of the 1,465 patients met these criteria.

Post‐stroke epilepsy was defined according to the ILAE diagnostic criteria for epilepsy^19^, ^21^ as the occurrence of at least one seizure ≥30 days after the stroke (which constitutes a high recurrence risk) or ≥2 seizures ≥7 days (late seizures) after the stroke, during the follow‐up of the first year from AIS onset (PSE group). The latter definition of PSE reflects the operational definition of epilepsy from ILAE^21^ and our clinical practice and was adopted in a previous study.^22^ The clinical events were evidenced by medical records (must include EEG findings).

Acute ischemic stroke patients without seizures during the follow‐up period were included in the no‐epilepsy AIS group.

The screening resulted in the identification of 312 AIS patients based on the inclusion criteria. 20 healthy controls from the medical examination center of our hospital were identified, with the age range (55–80 years). The participants with any known neurological condition or other diseases were excluded. This sample was necessarily much smaller than the AIS group due to the lower numbers of healthy individuals who are scanned at the hospital.

### 2.3 Baseline AIS characteristics

Medical records of the enrolled patients were reviewed for the relevant data, including age, sex, and National Institute of Health stroke scale (NIHSS) on admission. Causes of AIS were categorized as large‐artery atherosclerosis, cardioembolism, small vessel occlusion, or other determined or undetermined causes. AIS treatments included anticoagulation or antiplatelet aggregation, intravenous thrombolysis, mechanical thrombectomy (MT), and bridged endovascular therapy with MT following thrombolysis. Based on the imaging findings, anatomical locations and the number of infarct lesions were recorded.

### 2.4 PVS scores and asymmetry

**FIGURE 1:**
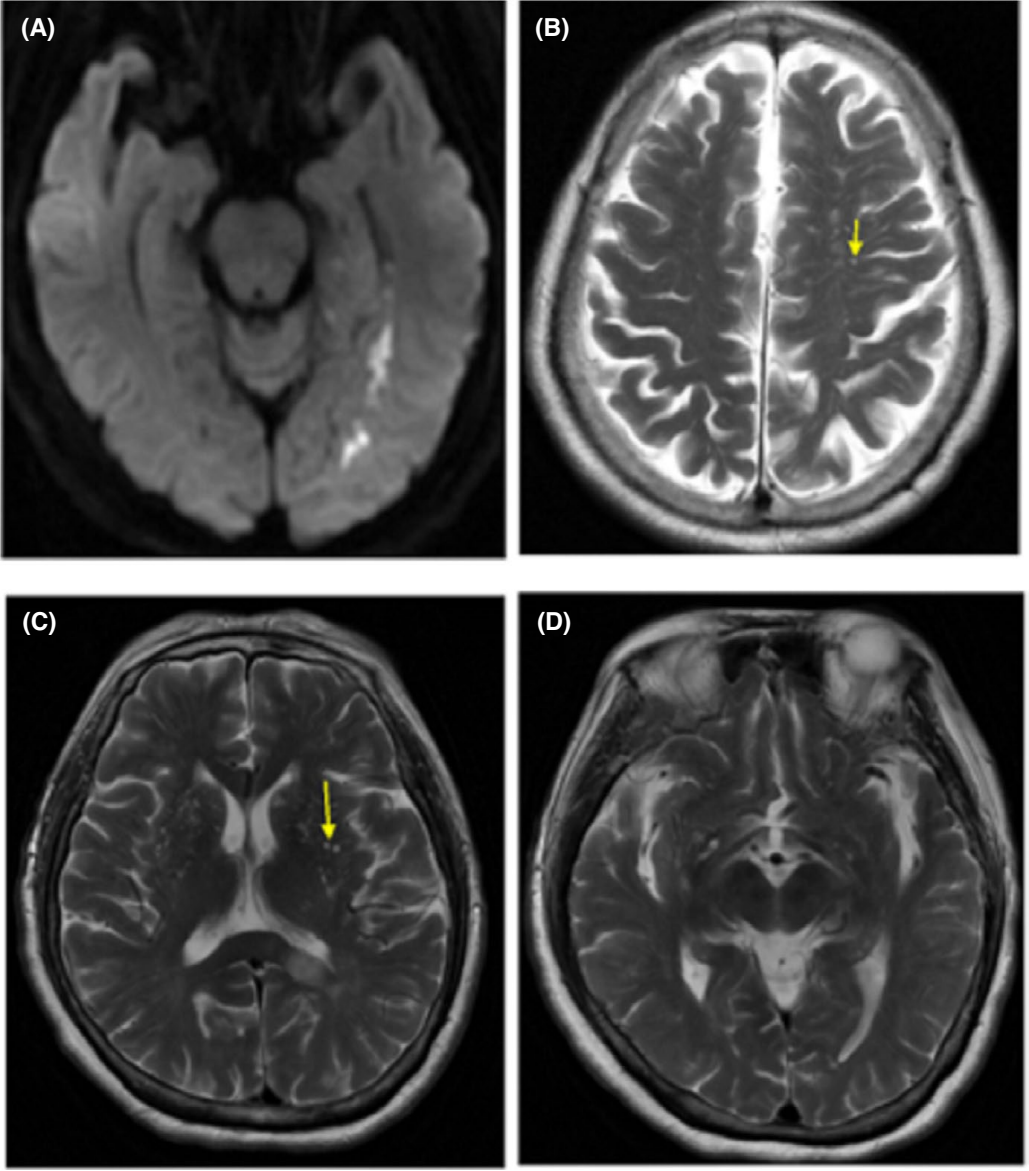
An example of PVS distribution in a patient with post‐stroke epilepsy. This was a 65‐year‐old woman presenting with retarded response to callings. She experienced an unprovoked seizure 7 months later. (A) Axial DWI showed acute infarction of left mesial temporal lobe. (B) Axial T2 showed a significantly asymmetric distribution of PVS in CS with more in left side. A similar effect was not observed in BG (C) and MB (D). BG, basal ganglia; CS, centrum semiovale; MB, midbrain; PSE, post‐stroke epilepsy

Perivascular spaces were segmented manually by author N.Y, a neuroradiologist, with 5‐year post‐qualification experience. PVSs were assessed on T_2_‐weighted MRI scans (resolution =1 × 1 × 6 mm, TR/TE/FA =7,411 ms/106 ms/90) acquired using a 3T MRI scanner (Siemens Verio) within 2 weeks after symptom onset of AIS.^23^ The total sequences of T1, T2, DWI, and FLAIR were used to differentiate and quantify PVS. EPVSs were defined as tubular linear when parallel or round ovoid dot‐like structures were perpendicular to the imaging plane with a CSF‐like signal intensity (hyperintense on T2‐weighted images) and a diameter of <3 mm. Different from EPVS, lacunar infarction is usually between 3 mm and about 15 mm in diameter and has a central CSF‐like hypointensity with a surrounding rim of hyperintensity on FLAIR images, which EPVS lacks.^24^ Figure 1 shows the examples of EPVS. For testing the inter‐rater reliability of the method, MRI was initially reviewed by two trained raters (N.Y.and L.M.G. Pa radiologist with 2 years of experience) blinded to clinical details. Discrepant ratings were reviewed by a third experienced rater (M.L.).

Enlarged PVSs in the infarct and contralateral hemispheres, or left and right for healthy controls were systematically assessed at three locations: basal ganglia (BG), centrum semiovale (CS), and midbrain (MB). The numbers of EPVS were graded as previously described^25^: for BG and CS, 0 = No, 1 = 1–10 EPVS (mild), 2 = 11–20 EPVS (moderate), 3 = 21–40 EPVS (frequent), 4=>40 EPVS (severe); for MB, 0 = No EVPS visible and 1 = EPVS visible. All relevant three slices for each location were reviewed, and the slice with the highest number of EPVS for the corresponding location was used for counting.

The assessment of EPVS asymmetry was modified from previously described methods.^17^, ^18^ The difference between the right and left side at each location was calculated as an asymmetry index (AI):

${\mathrm{AI}}_{J}=\left|\frac{s_{\mathrm{JR}}‐s_{\mathrm{JL}}}{s_{\mathrm{JR}}+s_{\mathrm{JL}}}\right|$, with 0≤AI≤1, J = region, L/R = left/right.where S_JL_ *and* S_JR_ are the number of EPVS in the left and right sides of the same observed region.

The total number of PVS of whole brain (S_T_) was defined the sum of the EPVS values in the three locations:

S_T_ = S_BG_+ S_CS_+ S_MB_.

EPVS AI of the whole brain (AI_T_) was calculated as follows:

${\mathrm{AI}}_{T}=\left|\frac{s_{\mathrm{TR}}‐s_{\mathrm{TL}}}{s_{\mathrm{TR}}+s_{\mathrm{TL}}}\right|$, with 0≤AI≤1.

A higher AI value implies more asymmetric distribution of EPVS in the brain. As an unbalanced distribution of EPVS at some levels may be observed in healthy controls,^17^, ^18^ we used a threshold of AI ≥0.2 to define a high asymmetry in EPVS distribution, in accordance with Duncan et al 2018,^18^ indicating that >60% of EPVSs were in one hemisphere. Based on this cutoff, an AI score of 0 (<0.2) and 1 (≥0.2) was assigned to each brain region.

### 2.5 Statistical analysis

To measure inter‐rater reliability, the second rater (L.M.G.P) counted EPVS on a subset of 30 cases (20 AIS patients and 10 controls) by the same method. The EPVSs of the remaining patients were counted by one rater (N.Y). The inter‐rater reliability was assessed using simple kappa for categorical data (EPVS score in MB), the weighted kappa test for ordinal data (EPVS score in BG and CS), and intraclass correlation coefficients (ICCs) for continuous data (EPVS numbers in BG and CS). For group comparisons, we used Student's *t*‐test for continuous variables, the chi‐squared test for categorical data, and the Wilcoxon rank‐sum test for ordinal variables. The Spearman rank‐order correlation coefficient was used to evaluate the strength and direction of associations of EPVS characteristics with age, sex, NIHSS, stroke causes, infarct location, and AIS treatments.

Multiple logistic regression (forward stepwise: likelihood ratio) was used to identify predictors of PSE. In this model, the presence of PSE was the dependent variable, and EPVS characteristics, age, gender, NIHSS, stroke causes, infarct location, and AIS treatments, were included as independent variables. Purposeful variables in the logistic model were selected based on the unique variable test with *p* < 0.10 ahead. Multicollinearity was assessed between the independent variables using a linear regression method. All statistical analyses were performed using the SPSS, Version 16. *p* < 0.01was considered statistically significant, but if there were multiple comparisons between three or more subgroups, a *p*‐value of <0.01/*N* after the Bonferroni correction was used to indicate the statistical significance (α_B_) to avoid type 1 error, where *N* was the number of subgroups.

## 3 RESULTS

### 3.1 Inter‐rater agreement on EPVS numbers and scores

A total of 30 individuals from the AIS patients (*n* = 312) and healthy controls (*n* = 20) were evaluated by both raters to assess inter‐rater reliability. Overall, there were moderate‐to‐good inter‐rater agreements on the measurement for EPVS scores and numbers. The kappa values for EPVS scores ranged from 0.50 to 0.92 in the various brain regions examined, and the ICC for EPVS numbers ranged from 0.63 to 0.96. The kappa value was 0.77 (95% confidence interval [CI] 0.70–0.83) for right MB, 0.75 (95% CI 0.68–0.81) for left MB, 0.61 (95% CI 0.50–0.71) for right BG, 0.84 (95% CI 0.77–0.92) for left BG, 0.73 (95% CI 0.63–0.82) for right CS, and 0.73 (95% CI 0.63–0.83) for left CS. There was also good inter‐rater agreement of the EPVS numbers between the two raters. The ICC and its 95% CI were 0.837 (0.75–0.90) for right BG, 0.94 (0.90–0.96) for left BG, 0.77 (0.69–0.85) for right CS, and 0.74 (0.63–0.82) for left CS, respectively.

### 3.2 EPVS characteristics between AIS patients and healthy controls

**FIGURE 2:**
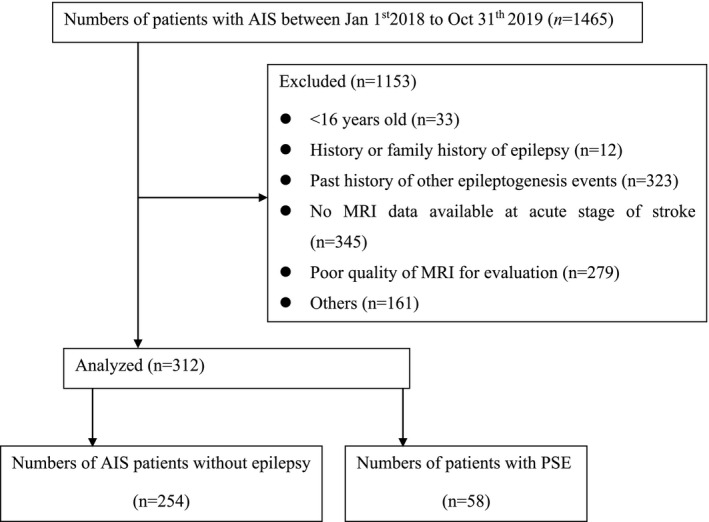
Consort diagram of excluded patients. AIS, acute ischemic stroke; PSE, post‐stroke epilepsy

Among 1,465 AIS patients during this study period, 312 AIS patients (120 females, 38.5%; mean age 67.46 ± 11.64 years) met the inclusion criteria. The consort diagram of the excluded patients is provided in Figure 2. The patients included were matched by 20 healthy participants (13 females, 65%) as the control group (mean age 61.00 ± 6.79 years; *p* = 0.015 compared with the AIS patients). The mean follow‐up time for all the enrolled AIS patients was 19.3 months (range 12.1–36.3 months). Both the EPVS number and scores were significantly higher in the BG and CS of the AIS group compared to those of the control group. No differences of EPVS numbers or scores were found in MB between the two groups. The AI score was significantly different in BG between the two groups (*p* < 0.01), but there was no difference in the AI scores of MB. There was no statistical difference of AI scores in the CS, though there was with the AI values (Table 1).

**TABLE 1:** Comparison of EPVS characteristics between AIS patients and healthy controls

Region			Basal ganglia			Centrum semiovale			Midbrain		
Group			Controls (*n* = 20)	AIS (*n* = 312)	*p*‐value	Controls (*n* = 20)	AIS (*n* = 312)	*p*‐value	Controls (*n* = 20)	AIS (*n* = 312)	*p*‐value
EPVS number (mean ± SD)	R		13.80 ± 4.05	24.78 ± 14.68	0.000	10.40 ± 3.84	21.38 ± 14.36	0.000	1.40 ± 1.23	0.98 ± 1.27	0.151
	L		13.90 ± 4.69	24.58 ± 14.43	0.000	9.80 ± 3.11	19.55 ± 12.85	0.000	1.60 ± 1.23	1.06 ± 1.28	0.069
EPVS scores	R	0	0	0	0.003	0	0	0.000	6 (30.0%)	156 (50.0%)	0.083
		1	4 (20.0%)	40 (12.8%)		14 (70.0%)	86 (27.6%)		14 (70.0%)	156 (50.0%)	
		2	14 (70.0%)	124 (39.7%)		6 (30.0%)	88 (28.2%)				
		3	2 (10.0%)	100 (32.1%)		0	104 (33.3%)				
		4	0	48 (15.4%)		0	34 (10.9%)				
	L	0	0	0	0.000	0	0	0.000	6 (30.0%)	148 (47.4%)	0.130
		1	6 (30.0%)	44 (14.1%)		16 (80.0%)	78 (25.0%)		14 (70.0%)	164 (52.6%)	
		2	12 (60.0%)	102 (32.7%)		4 (20.0%)	118 (37.8%)				
		3	2 (10.0%)	118 (37.8%)		0	94 (30.1%)				
		4	0	48 (15.4%)		0	22 (7.1%)				
AI			0.08 ± 0.07	0.18 ± 0.15	0.003	0.06 ± 0.07	0.18 ± 0.15	0.001	0.27 ± 0.41	0.45 ± 0.45	0.067
AI scores		0	20 (100%)	192 (61.5%)	0.001	18 (90%)	202 (64.7%)	0.021	12 (60%)	140 (44.9%)	0.189
		1	0	120 (38.5%)		2 (10.0%)	110 (35.3%)		8 (40.0%)	172 (55.1%)	

Note

EPVS scores in BG and CS: 0 = no EPVS visible; 1 = 1–10 EPVS (mild); 2 = 11–20 EPVS (moderate); 3 = 21–40 EPVS (frequent); 4 => 40 EPVS (severe); PVS scores in MB: 0 = no EVPS visible; 1 = one or more EVPS visible. *p* values were from the comparison with control group.

AI scores: 0 = AI < 0.2, 1 = AI ≥0.2.

Abbreviation: AI, asymmetric index; L, left; R, right.

### 3.3 Relevant factors for EPVS characteristics of AIS patients

In AIS patients, total EPVS numbers (BG, CS, and MB combined) increased with age (Pearson correlation, *r* = 0.22, *p* < 0.000). The scores and numbers of EPVS were not associated with stroke subtypes (duration time of symptoms before treatment, treatment methods after stroke, stroke causes stroke laterality, and infarct number) or severity (NIHSS at admission) (all *p* > 0.01).

The scores, numbers, and AI of EPVS at each observed region were not statistically different between in the unilateral stroke (*n* = 286) and bilateral stroke (*n* = 26) (all *p* > 0.01). Among the patients with unilateral stroke (*n* = 286), no significant differences of EPVS numbers (*p* = 0.61 in BG; *t* = 0.77, *p* = 0.44 in CS; *t* = 1.47, *p* = 0.14 in MB) and scores (*p* = 0.12 in BG and *p* = 0.24 in CS by the Kolmogorov‐Smirnov test; *p* = 0.28 in MB by likelihood ratio) were found between the infarct side and the noninfarct side.

### 3.4 Clinical characteristics between AIS without epilepsy and with PSE

Fifty‐eight (18.59%) of the 312 patients developed PSE with a median time of 36 days after stroke (range: 8 days‐304 days). The mean follow‐up time was 16.3 months (range: 12.2–25.1 months) for PSE patients and 19.2 months (range: 12.1–36.3 months) for the AIS patients without epilepsy. As shown in Table 2, compared to the nonepilepsy AIS group, the PSE patients were younger, had higher NIHSS at admission, higher proportions of cortical lesions, and higher proportions of large‐artery atherosclerosis and cardioembolic strokes. There were no differences in sex distribution, stroke laterality, and infarct numbers between the AIS without epilepsy and PSE patients.

**TABLE 2:** Clinical characteristics of nonepilepsy AIS and PSE patients

	Nonepilepsy AIS (*n* = 254)	PSE (*n* = 58)	*p*‐value	α_B_
Age (years)	68.29 ± 12.00	63.83 ± 9.16*	0.002	
Gender				
Male	152 (59.8%)	40 (69.0%)	0.232	
Female	102 (40.2%)	18 (31.0%)		
Duration of symptoms (days)	3.11 ± 3.46	3.06 ± 3.08	0.914	
NIHSS at admission*				
0	8 (3.1%)	0 (0%)	0.000	
1–4	30 (11.8%)	0 (0%)		
5–15	118 (46.5%)	22 (39.9%)		
16–20	70 (27.6%)	18 (31.0%)		
21–42	28 (11.0%)	18 (31.0%)		
Stroke laterality				
Left	148 (58.3%)	30 (51.7%)	0.045	0.003
Right	90 (35.4%)	18 (31.0%)		
Both	16 (6.3%)	10 (17.2%)		
Infarct number				
Unifocal	150 (59.1%)	26 (44.8%)	0.049	
Multifocal	104 (40.9%)	32 (55.2%)		
Stroke region				
Single‐lobe cortical	26 (10.2%)	18 (31.0%)*	0.000	
Cerebellum	12 (4.7%)	2 (3.4%)		
Brainstem	34 (13.4%)	0 (.0%)		0.000
BG	50 (19.7%)	10 (17.2%)		
CS	82 (32.3%)	2 (3.4%)		
Multilobe cortical	46 (18.1%)	22 (37.9%)*		
PC+AC	4 (1.6%)	4 (6.9%)		
Stroke causes				
Large artery atherosclerosis	74 (29.1%)	30 (51.7%)*	0.000	
Cardioembolism	24 (9.4%)	12 (20.7%)		0.000
Small‐vessel occlusion	138 (54.3%)	8 (13.8%)		
Other causes	18 (7.1%)	8 (13.8%)		
AIS treatment				
Anticoagulated or platelet therapy	194 (76.4%)	40 (69.0%)	0.172	
Thrombolysis	16 (6.3%)	8 (13.8%)		0.003
MT	26 (10.2%)	4 (6.9%)		
Thrombolysis and MT	18 (7.1%)	6 (10.3%)		

Abbreviations: BG, basal ganglia; CS, centrum semiovale; MT, mechanical thrombectomy; PC+AC, posterior circulation and anterior circulation stroke; PSE, post‐stroke epilepsy.

* *p* < 0.05, VS nonepilepsy AIS group. α_B_ = 0.01/*N* for Bonferroni correction.

### 3.5 EPVS characteristics between the AIS without epilepsy and PSE patients

As shown in Table 3, there were no significant differences in EPVS scores of bilateral BG, CS, and MB between the PSE group and the nonepilepsy AIS group. There were also no significant differences on the total EPVS numbers and AI values between the PSE group and the nonepilepsy AIS group. The marked differences of AI score in the CS and midbrain region were found between the PSE group and the nonepilepsy AIS group (*p* = 0.004). There was no statistical difference of AI scores in the MB for the PSE and nonepilepsy AIS groups, though there was with the AI values. The AI score was not significantly different between groups in the other brain regions, or in the whole brain.

**TABLE 3:** Comparison of EPVS characteristics between patients with and without post‐stroke epilepsy

Region			Basal ganglia			Centrum semiovale			Midbrain		
Group			Nonepilepsy AIS (*n* = 254)	PSE (*n* = 58)	*p*‐value	Nonepilepsy AIS (*n* = 254)	PSE (*n* = 58)	*p*‐value	Nonepilepsy AIS (*n* = 254)	PSE (*n* = 58)	*p*‐value
EPVS number	R		25.40 ± 15.00	22.03 ± 12.98	0.115	21.10 ± 14.41	22.59 ± 14.14	0.478	1.01 ± 1.27	0.86 ± 1.23	0.429
	L		25.08 ± 14.34	22.41 ± 14.76	0.205	19.65 ± 13.17	19.14 ± 11.42	0.786	1.11 ± 1.31	0.86 ± 1.11	0.183
EPVS scores	R	0	0	0	0.025	0	0	0.489	124 (48.8%)	32 (55.2%)	0.383
		1	32 (12.6%)	8 (13.8%)		70 (27.6%)	16 (27.6%)		130 (51.2%)	26 (44.8%)	
		2	92 (36.2%)	32 (55.2%)		76 (29.9%)	12 (20.7%)				
		3	90 (35.4%)	10 (17.2%)		82 (32.3%)	22 (37.9%)				
		4	40 (15.7%)	8 (13.8%)		26 (10.2%)	8 (13.8%)				
	L	0	0	0	0.030	0	0	0.195	116 (45.7%)	32 (55.2%)	0.359
		1	36 (14.2%)	8 (13.8%)		68 (26.8%)	10 (17.2%)		138 (54.3%)	26 (44.8%)	
		2	74 (29.1%)	28 (48.3%)		94 (37.0%)	24 (41.4%)				
		3	104 (40.9%)	14 (24.1%)		72 (28.3%)	22 (37.9%)				
		4	40 (15.7%)	8 (13.8%)		20 (7.9%)	2 (3.4%)				
AI			0.17 ± 0.15	0.20 ± 0.14	0.305	0.17 ± 0.15	0.24 ± 0.17	0.001	0.49 ± .46	0.30 ± 0.40	0.002
AI scores		0	160 (63.0%)	32 (55.2%)	0.269	178 (70.1%)	24 (41.4%)	***0.000***	106 (41.7%)	34 (58.6%)	0.020
		1	94 (37.0%)	26 (44.8%)		76 (29.9%)	34 (58.6%)		148 (58.3%)	24 (41.4%)	

Note

Bold italics values indicate *p* < 0.001.

EPVS scores for BG and CS: 1 = 1–10 EPVS (mild); 2 = 11–20 EPVS (moderate); 3 = 21–40 EPVS (frequent); 4 => 40 EPVS (severe); EPVS scores for MB: 0 = no EVPS visible; 1 = no EVPS visible.

AI scores: 0 = AI < 0.2, 1 = AI ≥0.2.

Abbreviation: AI, asymmetric index.

### 3.6 A multivariate model for predicting PSE

Post‐stroke epilepsy was predicted by a binary logistic regression model (Odds ratio 0.228, *Nagelkerke R^2^* = 0.518, *p* < 0.001) (Table 4) that included 5 variables with univariate significance of *p* < 0.05: the presence of age, stroke cause, NIHSS at admission, single‐lobe cortical involvement of stroke regions, and high EPVS asymmetry in CS (OR = 3.709, CI [1.508–9.123], *p* = 0.004). The percentage accuracy in classification is 81.4%. The sensitivity of the model including 4 parameters as listed in Table 4 for predicting PSE was 93.7%, and the specificity was 48.3%.

**TABLE 4:** Predictors for PSE analyzed by the binary logistic regression

	β	Wald	*p*‐value	Odds ratio 95% (CI)
Age	−0.058	9.923	0.002	0.944 (0.910–0.978)
Stroke causes				
Other causes	1.186	15.086	0.002	1
Large artery atherosclerosis	−3.091	9.295	0.002	0.045 (0.006–0.332)
Cardioembolism	−3.094	7.957	0.005	0.045 (0.005–0.389)
Small‐vessel occlusion	−4.342	13.402	0.000	0.013 (0.001–0.133)
NIHSS score at admission				
21–42		3.076	0.545	
0	−21.127	.000	0.999	0.000 (0.000)
1–4	−21.698	.000	0.997	0.000 (0.000)
5–15	−1.186	2.898	0.089	0.305 (0.078–1.196)
16–20	−0.867	2.103	0.147	0.420 (0.130–1.357)
Stroke regions				
PC+AC	**‐**	18.146	0.006	1
Single‐lobe cortical	1.973	3.502	0.061	7.191 (0.911–56.778)
Cerebellum	−0.600	0.174	0.677	0.549 (0.033–9.200)
Brainstem	−22.359	0.000	0.997	0.000 (0.000–0)
BG	1.437	1.050	0.305	4.209 (0.269–65.751)
CS	−1.318	0.918	0.338	0.268 (0.018–3.967)
Multilobe cortical	−0.221	0.068	0.794	0.802 (0.153–4.210)
CS AI score	1.311	8.149	0.004	3.709 (1.508–9.123)

As listed in Table 5, it can be preliminarily considered that the multicollinearity interaction can be ignored due to all *tolerance* <*0*.*1* and *variance inflation factor* <5 for each independent variable (*r^2^* = *0*.*161*, *F* = *11*.*78*, *p* < 0.001).

**TABLE 5:** Multicollinearity analysis for independent variables in logistic regression model

	Unstandardized coefficients		Standardized coefficients	t	Sig.	Collinearity statistics	
	B	S.E.	Beta			Tolerance	VIF
(Constant)	1.042	0.172		6.065	0.000		
Age at stroke	−0.005	0.002	−0.149	−2.773	0.006	0.945	1.059
NIHSS scores	0.078	0.023	0.189	3.399	0.001	0.891	1.123
Stroke causes	−0.014	0.023	−0.036	−.606	0.545	0.780	1.283
CS AI scores	0.101	0.045	0.124	2.247	0.025	0.895	1.117
Stroke region	0.059	0.016	0.221	3.688	0.000	0.762	1.312

Abbreviation: VIF, variance inflation factor.

## 4 DISCUSSION

Our study demonstrated that an asymmetric distribution, but not the number, of PVS in CS during the acute ischemic stroke period was independently associated with the development of PSE. This implies that PSE was associated with an imbalance, rather than the absolute amount, of EPVS in CS. Asymmetric distributions of EPVS in other brain regions (MB and BG) and the amount of PVS in each brain region (BG, CS, or MB) did not show similar association.

There is a lack of experimental data to explain the potential mechanisms of this observation. We speculate that it may be related to the much larger area of CS with more EPVS adjacent to the cortex than other regions of BG and MB. This might lead to asymmetry in CSF drainage of metabolites with subsequent metabolic and electrophysiologic imbalance of the brain, resulting in seizures. In line with this hypothesis, asymmetry of EEG background activity has been observed as an independent predictor of PSE during the first year after stroke.^26^

Another potential explanation for the association between EPVS and PSE may be the relationship of the shared signaling pathways between epilepsy‐related immunological inflammation in the brain (which could induce epilepsy and also be induced by epilepsy) and EPVSs. Growing experimental studies and clinical evidence have demonstrated that inflammatory reactions in the brain can increase the permeability of the blood‐brain barrier (BBB) to proinflammatory molecules and cells and enhance neuronal excitability to trigger seizures.^27^ Impaired PVS could allow leukocytes and antigen‐presenting cells penetrate the glia, then releasing proinflammatory molecules further degrade BBB structures, which rarely occurs under basal conditions.^28^ Furthermore, EPVSs are proposed to form part of a complex brain fluid drainage system to support interstitial fluid exchange and facilitate clearance of waste products from the brain. Impaired function of the PVSs may further lead to reduced blood flow, oxidative stress, hypoperfusion, and hypoxia, which are linked to PSE.^29^

Moreover, Gaberel et al^30^ have reported that the PVSs as glymphatic system were initially decreased and impaired around the infarct lesion in acute phase of 3 h after embolic ischemic stroke in mice, possibly due to its blocking effect of infarct lesion; however, this effect was reversed to the baseline level after 24 h of stroke, possibly due to its spontaneous arterial recanalization. In another experimental study, increased PVSs were observed at 48 h in a rat model of cerebral ischemia‐reperfusion injury.^31^ We also found this phenomenon of decreased PVSs around the infarct lesion in several cases of AIS within 24 h but totally with no statistical significance on the EPVS scores and counts between the infarct side and the noninfarct side. Maybe more rigors of study design on the onset phase were needed for this interesting question.

As many previous studies have reported,^1^, ^3^, ^4^ our study also showed that cortical involvement, carotid circulation territory with large arteries, and stroke severity at admission are independent risk factors for PSE. However, there was no significant association between the predictors above and the EPVS‐related parameters in our AIS patients. Previous studies have examined the impact of AIS treatments on PSE or EPVSs, with conflicting findings.^32^, ^33^, ^34^ Our current study also examined the effects of the different treatments with t‐PA, anticoagulated or antiplatelet aggregation, and bridged MT following thrombolysis on the development of PSE. But there were no significant differences of the associations with the above treatments observed on the PVS characteristics or epileptogenesis after stroke.

Potential clinical implications of PVS involvement in other stroke outcomes have also been studied. Liang et al.^14^ showed that the PVS scores in CS were associated with post‐stoke depression at 3 months after AIS. Others have reported higher PVS scores in the BG to be associated with poorer neurological function, greater disability, and poorer quality of life in AIS patients after the follow‐up of 3–6 months.^35^ The presence of more numerous PVS was shown to be associated with the leukoaraiosis and hemorrhagic transformation, poor physical outcome, cognitive decline, and stroke recurrence in AIS.^10^

The underlying causes of EPVS have not been fully elucidated. However, several common risk factors for EPVS have been established, such as old age, diabetes, and hypertension, whereas high BMI and alcohol were considered its protective factors.^36^, ^37^ A recent meta‐analysis including 23 relevant studies showed that higher EPVS prevalence was associated with aging, hypertension, more lacunes, and microbleeds but not white‐matter hyperintensities, stroke, or cognitive impairment.^38^ Consistently with these observations, our study found increased EPVS in AIS compared with healthy controls; and in the AIS patients, increased EPVS was found in older age, but not associated with other various stroke parameters, or the treatment after stroke.

Our study has limitations. Firstly, not all the AIS patients were enrolled in the study, due to the limitation of MR acquirement at their acute stage. Secondly, the health control group was relatively smaller and younger, which was due to relatively fewer healthy people undergoing brain MRI. Thirdly, the other limitation for this study may be the method of counting EPVS manually, with potential subjectivity, although good inter‐rater agreement was demonstrated. We also do not know whether and when the asymmetric distribution of EPVS would resolve over time, or whether it existed before the stroke. The latter would imply certain individuals may be intrinsically more prone to develop PSE. Lastly, the AI of EPVS did not contain the factor of PVS diameter. It is worth using the diameter of PVS to quantify asymmetry by some software in our future studies.

Our present findings suggest that asymmetric distribution of EPVSs in the CS may be a novel imaging biomarker for the development of PSE. An early predictor for PSE will provide better evidence and choice for early antiepileptic treatment. The AI score of EPVS provides a novel imaging biomarker for the understanding of epileptogenesis after stroke. Further studies should aim to explain the mechanism of this association and to confirm the association with larger patient populations.

## 5 CONCLUSIONS

This study explored the factors influencing EPVS characteristics at the onset time of first AIS, and whether it could predict PSE. The EPVS scores were significantly higher in the AIS patients compared to those of health controls. Higher asymmetry index scores of EPVS in CS were found between the PSE and nonepilepsy AIS groups. Interestingly, this asymmetric distribution of EPVS was not found in other brain regions between the PSE and nonepilepsy AIS groups. An asymmetric distribution of EPVS in CS may be an independent risk factor or a novel imaging biomarker for PSE. Future studies should explore the relationship between EPVS and seizure types, seizure frequency, EEG findings, and AED treatment outcome.

## CONFLICTS OF INTEREST

The authors declare that the research was conducted in the absence of any commercial or financial relationships that could be construed as a potential conflict of interest.

## Data Availability

The authors confirm that the data supporting the findings of this study are available within the article after deidentification (text, tables, and figures). All the original data in statistics of this study can be got from the corresponding authors, upon reasonable request at any time. We confirm that we have read the Journal's position on issues involved in ethical publication and affirm that this report is consistent with those guidelines. The studies involving human participants were reviewed and approved by Nanjing Brain Hospital affiliated to Nanjing Medical University. All participants or their family members or authorized legal representatives provided written informed consent.
